# Survival prognostic factors for differentiated thyroid cancer patients with pulmonary metastases: A systematic review and meta-analysis

**DOI:** 10.3389/fonc.2022.990154

**Published:** 2022-12-15

**Authors:** Hao Zhao, Chun-Hao Liu, Yue Cao, Li-Yang Zhang, Ya Zhao, Yue-Wu Liu, Hong-Feng Liu, Yan-Song Lin, Xiao-Yi Li

**Affiliations:** ^1^ Department of General Surgery, Peking Union Medical College Hospital, Chinese Academy of Medical Sciences & Peking Union Medical College, Beijing, China; ^2^ Department of Nuclear Medicine, Peking Union Medical College Hospital, Chinese Academy of Medical Sciences & Peking Union Medical College, Beijing, China

**Keywords:** differentiated thyroid cancer, pulmonary metastases, prognosis, meta-analysis, systematic review

## Abstract

**Background:**

The prognostic factors for differentiated thyroid cancer (DTC) patients with pulmonary metastases (PM) remain scantly identified and analyzed. Therefore, this systematic review and meta-analysis were performed to identify and summarize the prognostic factors in adult DTC patients with PM to help distinguish patients with different prognoses and inform the rational treatment regimens.

**Method:**

We performed a comprehensive search of the relevant studies published in the Cochrane Library, PubMed, Scopus, Embase, Wanfang database, VIP database, China National Knowledge Infrastructure, and Google Scholar from their inception until February 2021. The pooled hazard ratios (HR) for overall survival and/or progression-free survival (PFS) with 95% confidence intervals were applied to evaluate and identify the potential prognostic factors. Pooled OS at different time points were also calculated for the available data. A random-effects model was used in the meta-analysis.

**Results:**

The review and meta-analysis included 21 studies comprising 2722 DTC patients with PM. The prognostic factors for poor OS were: age over 40 years (HR=7.21, 95% confidence interval [CI] 1.52-34.10, P=0.01, N=788), age over 45 years (HR=2.18, 95% CI 1.26-3.77, P<0.01, N=601), male gender (HR=1.01, 95% CI 1.01-1.19, P=0.03, N=1396), follicular subtype of thyroid cancer (HR=1.63, 95% CI 1.36-1.96, P<0.01, N=2110), iodine non-avidity (HR=3.10, 95% CI 1.79-5.37, P<0.01, N=646), and metastases to other organs (HR=3.18, 95% CI 2.43-4.16, P<0.01, N=1713). Factors associated with poor PFS included age over 45 years (HR=3.85, 95% CI 1.29-11.47, P<0.01, N=306), male gender (HR=1.36, 95% CI 1.06-1.75, P=0.02, N=546), iodine non-avidity (HR=2.93, 95% CI 2.18-3.95, P<0.01, N=395), pulmonary metastatic nodule size over 10mm (HR=2.56, 95% CI 2.02-3.24, P<0.01, N=513), and extra-thyroidal invasion (HR=2.05, 95% CI 1.15-3.67, P=0.02, N=271). The pooled 1, 3, 5, 10, 15, and 20-years OS were 95.24%, 88.46%, 78.36%, 64.86%, 56.57%, and 51.03%, respectively.

**Conclusions:**

This review and meta-analysis identified the prognostic factors of DTC patients with PM. Notably, FTC, metastases to other organs, and iodine non-avidity were particularly associated with poor prognosis. The identified prognostic factors will help guide the clinical management of DTC patients with PM.

**Systematic review registration:**

https://inplasy.com/inplasy-2022-2-0026/, identifier (INPLASY202220026).

## 1 Introduction

The past decades have witnessed a rapid increase in the global incidence of thyroid cancer. As the most common endocrine malignancy, thyroid cancer ranks ninth among all cancers in incidence (586,000 cases worldwide), with an increasing trend (1, 2). Over 90% of thyroid cancers are differentiated thyroid cancers (DTC), characterized by an excellent prognosis with a 10-year survival rate exceeding 90% (3, 4). Nevertheless, the survival rate of DTC drops significantly upon the occurrence of distant metastases (DM) (5, 6). The Surveillance, Epidemiology, and End Results (SEER) database reported a 76% and 64% 5-year overall survival (OS) rates for papillary thyroid cancer (PTC) and follicular thyroid cancer (FTC) with DM, respectively (7). DM is, thus, a significant prognostic factor for the survival of DTC patients.

Though DTCs with DM have an overall poor prognosis, there are still significant variations between subgroups. Studies postulate that the 10-year OS rate varies from 10% to 90% for different subgroups (8). For example, the 10-year OS rate for radioiodine avidity patients can reach 55% (9–11), while non-avidity patients have an OS of 10-18% (9, 12, 13). Meanwhile, treatment strategies for different subgroups, including the management options for localized lesions and optimal time to start systemic treatment like tyrosine kinase inhibitor (TKI) therapy, remain controversial (14). Therefore, identifying the prognostic factors of these patients could help with risk stratification and formulating treatment plans for different subgroups, thereby improving the long-term prognosis.

Distant metastases of DTCs commonly occur in the lungs (85%), followed by the bones (39.9%), while its occurrence in the brain (5.8%) and liver (3.6%) is relatively rare (15). Several studies have identified factors related to DM occurrence in DTCs, including old age, male gender, pathological subtypes, large primary tumor sizes, and extrathyroidal extension (ETE). However, only a few studies have focused on the prognostic factors of DTC patients with DM, especially of DTC patients with pulmonary metastases (PM). Previous studies suggest these factors, together with metastases size, multiorgan metastases, thyroglobulin (Tg) level, BRAF^V600E^ mutation, radioiodine avidity, and TKI administration, among other factors, may affect the prognosis of DM (9, 10, 15–30). Deciphering the role and impact of these factors on prognosis is thus crucial in aiding treatment decision-making.

This systematic review and meta-analysis aimed to identify and summarize the prognostic factors in adult DTC patients with PM to help distinguish patients with different prognoses and inform the rational design of treatment regimens.

## 2 Materials and methods

### 2.1 Guidelines and protocols

This systematic review and meta-analysis were conducted following the statement (31), and guidelines for systematic reviews and meta-analyses of prognostic factors (32). The protocol used for this systematic review and meta-analysis is registered in INPLASY (https://inplasy.com/) with ID INPLASY202220026.

### 2.2 Literature search

Two reviewers (Z.H. and L.C.H.) performed independent, comprehensive searches of relevant studies published in the Cochrane Library, PubMed, Scopus, Embase, Wanfang database, VIP database, China National Knowledge Infrastructure (CNKI) from their inception to February 2021 following the registered protocol. We also retrieved citations and references in Google Scholar to ensure no relevant literature was missed. There was no restriction on language and time of publication to limit publication bias. No search was done for the unpublished data and grey literature. **Supplementary S1** outlines the detailed search strategy.

### 2.3 Study selection

We considered randomized clinical trials (RCTs), retrospective and prospective observation cohort studies for inclusion. Inclusion criteria were: studies enrolled mostly adult patients (≥18 years) with a confirmed diagnosis of DTC through histopathology and PM though chest CT, chest x-ray, or 131I whole-body scan (WBS); explored at least one prognostic factor for DTC patients with PM; reported OS and/or progression-free survival (PFS) as an outcome of interest; with an available English abstract and an accessible full text was required. Studies with fewer than 20 cases of DTC patients with PM, those impossible to extract or convert valid data, repeated published literature, case reports, reviews, conference reports, animal experiments, and *in vitro* cell experiments were excluded.

All the identified citations were imported into Rayyan (rayyan.qcri.org) to assess their eligibility after removing the duplicates. The two reviewers (Z.H. and L.C.H.) subsequently completed the screening process independently. A third reviewer (L.X.Y.) was invited to make the final decision if there was a disagreement between the two reviewers even after the discussion.

### 2.4 Data extraction and quality assessment

The researchers independently used standardized data extraction forms based on the CHARMS-PF checklist in Microsoft Excel to extract essential data (e.g., date, settings, study design, and prognostic factors) from eligible studies32. The data was subsequently reviewed, and all disputes were resolved by consensus based on discussion. Study authors were not contacted to obtain unpublished data.

The quality in prognosis studies (QUIPS) tool developed by Hayden et al. was used to assess the bias risk of included studies 33. This tool includes six domains. Studies with five or six low-risk domains were classified to have an overall low risk of bias, while those with two or more high-risk domains were classified as having an overall high risk of bias. The remaining studies were classified as having an overall moderate risk of bias. The assessment results of the risk of bias were summarized and presented graphically. An overall certainty assessment in pooled estimates was performed using the grading of recommendations, assessment, development, and evaluation (GRADE) approach adopted in prognostic studies.

### 2.5 Data synthesis

In this prognostic meta-analysis, the pooled HR of OS and/or PFS with 95% confidence intervals (CI) was calculated for potential prognostic factors to investigate the effects of the factors. If possible, an indirect extraction of the HR values was done when unreported using the method described by Perneger et al. 34. A meta-analysis of OS estimates was conducted in cases with sufficient data at different time points (at 1, 3,5, 10, 15, and 20 years as reported). The interstudy heterogeneity was accessed using I^2^ value. I^2^ ≤ 50% indicated low heterogeneity and the fixed-effects model was adopted, while I^2^>50% indicated significant heterogeneity and the random-effects model was adopted. If necessary, meta regression was performed to explore the potential source of heterogeneity. Evidence of publication bias was accessed by funnel plot analysis and Egger’s test if at least ten studies were included (present in the Supplementary). All analyses were performed using the “metafor” package in R version 4.0.2 (R Core Team, R Foundation for Statistical Computing, Vienna, Austria). The significance threshold was set at P<0.05 or a 95% CI excluding 1.00.

## 3 Results

### 3.1 Search results

#### 3.1.1 Identification of the relevant studies

**Figure 1:**
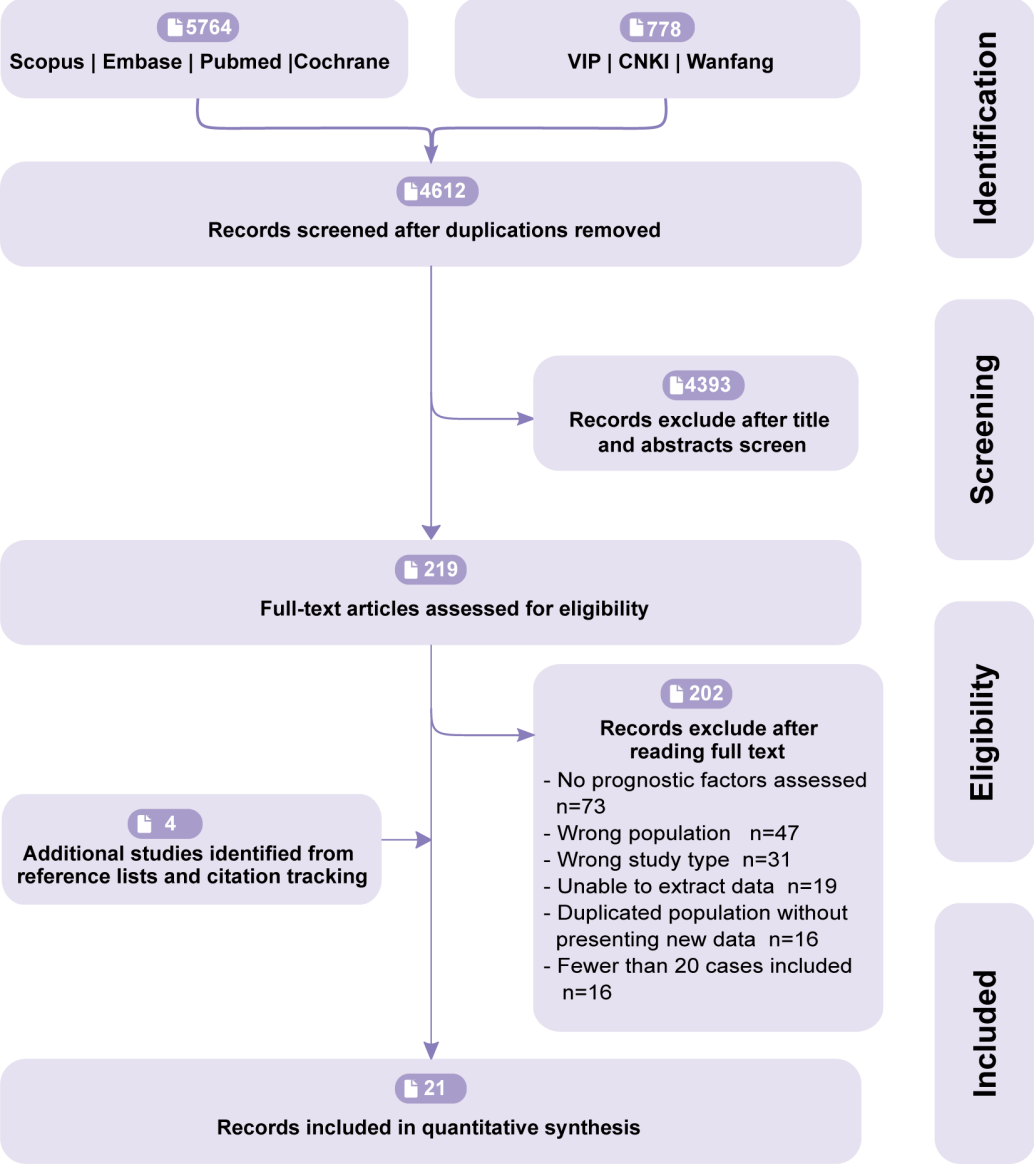
Flowchart of summarising reference search and study selection.

Our initial database search retrieved 6542 eligible studies. Exclusion of 1930 duplications, 4393 irrelevant studies based on the title and abstract, and 219 studies after full-text reading left 17 suitable studies. An additional four papers were identified from reference lists and citation tracking (**Figure 1**), totaling 21 studies comprising 2722 DTC patients with PM, which were included in the analysis.

#### 3.1.2 Characteristics of the included studies

**Supplementary Table 1** summarises the characteristics of the included studies. Of the 21 studies, 12 were from Asia, five were from Europe, three were from North America, and one was from South America. Most studies were published between 2010 and 2020. All studies were retrospective, while one was a multi-centered study.

#### 3.1.3 Quality of the included studies

The bias risk assessment of the included studies using the QUIPS tool was shown in **Supplementary Figure 1**. Most of the studies were low-intermediate risk (n=17, 80.95%), with only four studies classified as high risk. The four studies were judged to have a risk of bias in study attrition and confounding, statistical analysis, and reporting.

### 3.2 Synthesis results

**Table 1** outlines pooled hazard ratios, 95% CI, and GRADE level of certainty for potential prognostic factors. The results are based on different clinical endpoints.

**Table 1:** Prognostic factors and associated hazard ratio of survival.

Prognostic factors	Studies	Cases	HR (95% CI)	P value	I^2^ (%)	GRADE certainty*
OS						
Age >40 yrs.	3	788	7.21 (1.52-34.10)	0.01	100	Low
Age >45 yrs.	7	601	2.18 (1.26-3.77)	<0.01	86	Moderate
Male sex	11	1396	1.01 (1.01-1.19)	0.03	0	Low
Histology: FTC	12	2110	1.63 (1.36-1.96)	<0.01	77	Low
^131^I non-avidity	4	646	3.10 (1.79-5.37)	<0.01	81	Moderate
Metastases to other organs: yes	10	1713	3.18 (2.43-4.16)	<0.01	95	Moderate
Primary tumor size >40mm	2	96	1.28 (0.58-2.80)	0.54	0	Low
Lymph node metastasis: yes	3	510	0.68 (0.41-1.12)	0.13	0	Low
PFS						
Age >45 yrs.	2	306	3.85 (1.29-11.47)	<0.01	89	Low
Age >55 yrs.	2	136	1.30 (0.06-30.17)	0.87	68	Low
Male sex	4	546	1.36 (1.06-1.75)	0.02	0	Low
^131^I non-avidity	3	395	2.93 (2.18-3.95)	<0.01	12	Moderate
PM nodule size >10mm	4	513	2.56 (2.02-3.24)	<0.01	0	Moderate
Primary tumor size >40mm	2	196	1.41 (0.79-2.25)	0.24	0	Low
Lymph node metastasis: yes	4	526	0.94 (0.47-1.90)	0.84	0	Low
Multifocality	2	154	1.81 (0.77-4.25)	0.18	0	Low
Extra-thyroidal invasion	3	271	2.05 (1.15-3.67)	0.02	0	Low

*GRADE certainty: The estimates were categorised into one of four levels of certainty: high, moderate, low, or very low.

OS, overall survival; PFS, progress-free survival.

#### 3.2.1 Age

**Figure 2:**
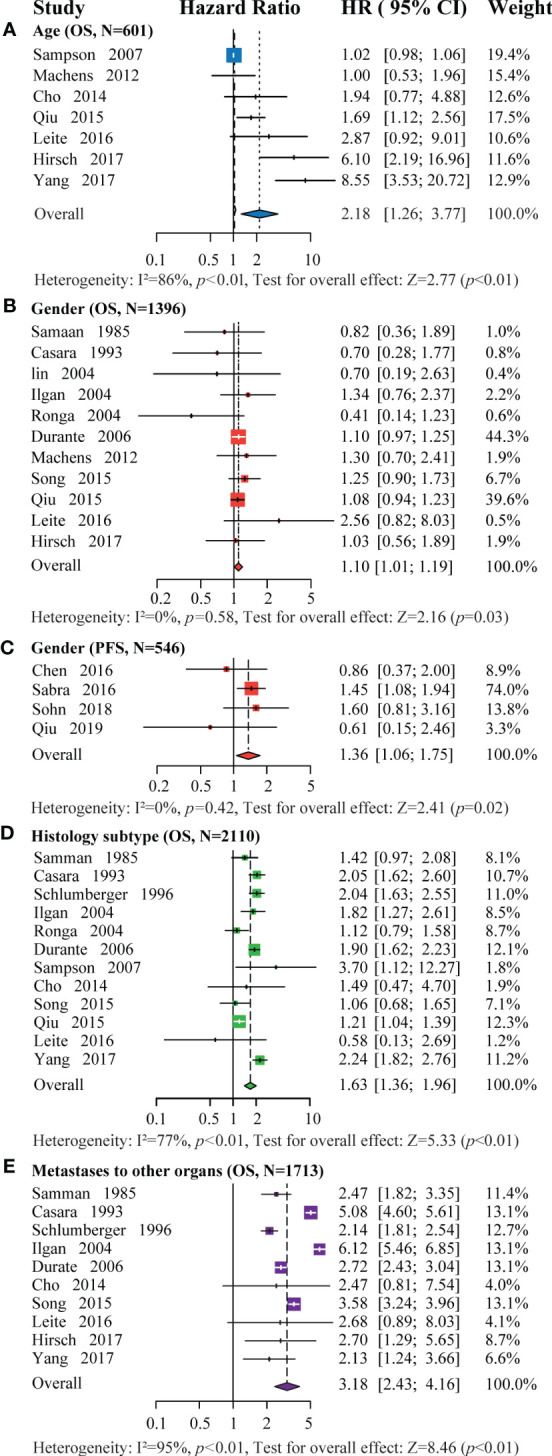
Forest plot for the association of: **(A)** age with OS (>45 years vs. ≤45 years), **(B)** gender with OS (male vs. female), **(C)** gender with PFS (male vs. female), **(D)** histology subtype with OS (FTC vs. PTC), and **(E)** metastases to other organs with OS (with vs. without).

**Figure 2A** shows the calculated pooled hazard ratios upon assessing the impact of age at four reported age thresholds. Patients under 45 years had a better OS than those over 45 years (HR=2.18 [CI 1.26-3.77], P<0.01, N=601, **Figure 2A**) (5, 15, 23, 27, 33–35). Similarly, patients under 40 years had a better OS than those over 40 years (HR=7.21 [CI 1.52 - 34.10], P=0.01, N=788, **Supplementary Figure 3**) (22, 25, 36). A single study reported that patients above 25 years had a poor OS (HR=2.36 [CI 1.67-3.04], P<0.01, N=42) (37). In the same line, the PFS was significantly better in the under-45 group than in the over-45 group (HR=3.85 [CI 1.29-11.47], P=0.02, N=306, **Supplementary Figure 4**) (20, 26). However, there was no significant difference in PFS between the under and over 55 groups (HR=1.30 [CI 0.06-30.17], P*=*0.87, N=136, **Supplementary Figure 5**) (24, 38).

#### 3.2.2 Gender

Eleven and four studies were included in OS and PFS analysis based on gender, respectively (10, 15, 19, 20, 22, 24–26, 33–39). Females had a significantly better OS than males (HR=1.01 [CI 1.01-1.19], P*=*0.03, N=1396, Fig 2B). Similarly, they had a significantly better PFS than males (HR=1.36 [CI 1.06-1.75], P*=*0.02, N=546, **Figure 2C**).

#### 3.2.3 Histology subtype

**Figure 3:**
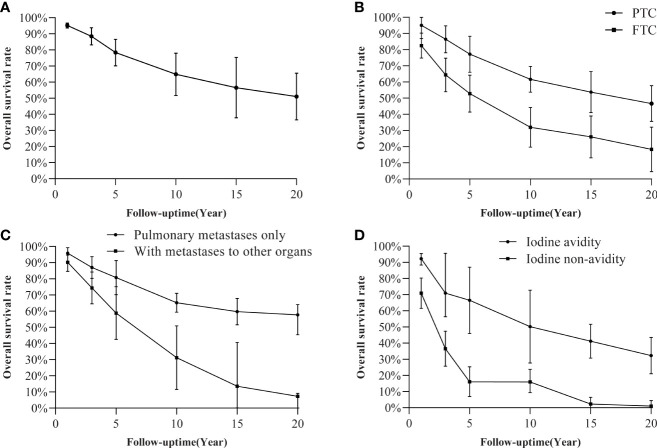
Pooled OS rate point estimates: **(A)** all included patients; **(B)** PTC versus FTC; **(C)** pulmonary metastases only versus with metastases to other organs; **(D)** iodine avidity versus iodine non-avidity.

Twelve studies comprising 1738 DTC patients with PM were included for histology subtype analysis (5, 9, 10, 19, 22, 23, 25, 27, 33, 34, 36, 37). The FTC group had a significantly poor OS than the PTC group (HR=1.63 [CI 1.36-1.96], P<0.01, N=2110, **Figure 2D**), consistent with the trend of the pooled OS (**Figure 3B**). However, a single study reported an insignificant difference in PFS between the two groups (HR=1.32 [CI 0.54-3.21], P=0.541, N=107, **Supplementary Table 2**) (20).

#### 3.2.4 Metastases to other organs

Ten studies comprising 1713 patients with DM were included in this meta-analysis (5, 9, 15, 19, 22, 25, 27, 34, 36, 37). DTC patients with metastases to other organs had a poorer OS than patients with PM alone (HR=3.18 [CI 2.43-4.16], P<0.01, N=1713, **Figure 2E**). This trend was similar to that of pooled OS (**Figure 3C**). Of note, one study also reported a poorer PFS in patients with metastases to other organs than those with PM only (HR=2.38 [CI 1.1-5.15], P=0.028, N=107) (20).

#### 3.2.5 Iodine avidity

**Figure 4:**
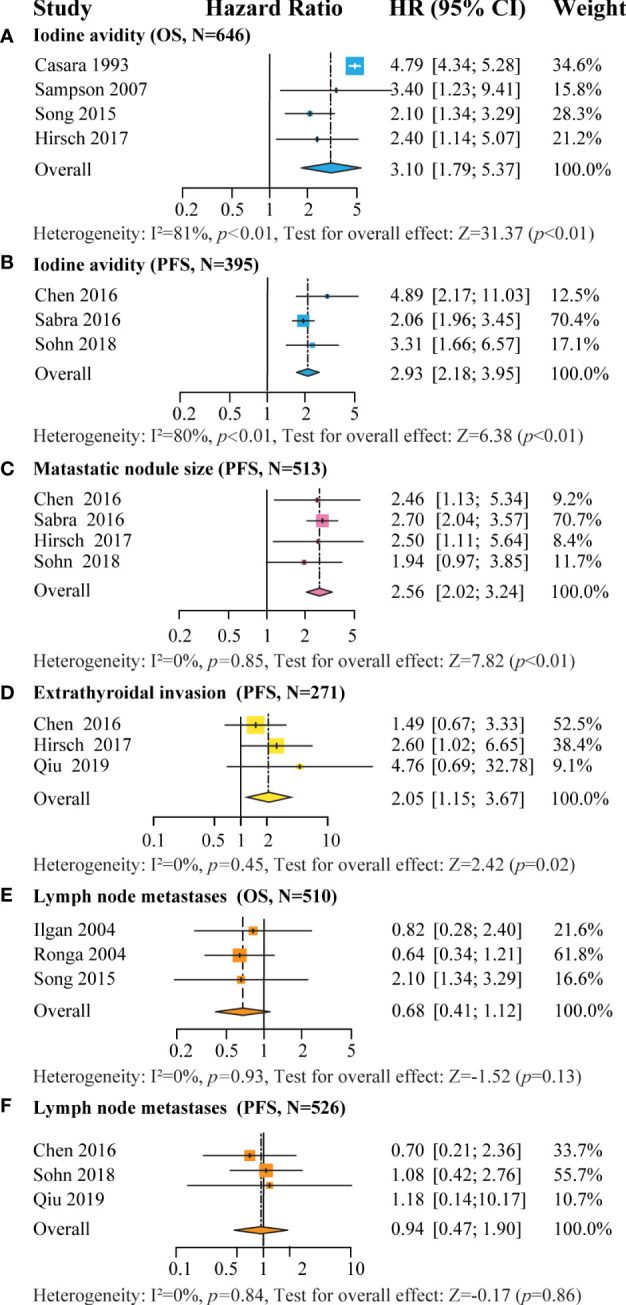
Forest plot for the association of: **(A)** iodine avidity with OS (non-avidity vs. avidity), **(B)** iodine avidity with PFS (non-avidity vs. avidity), **(C)** metastatic nodule size with PFS (≥ 10mm vs. <10mm), **(D)** extra thyroid invasion with PFS (with vs. without), **(E)** LNM with OS (with vs. without), **(F)** LNM with PFS (with vs. without).

Seven studies were included in the meta-analysis of iodine avidity: four studies for OS and three studies for PFS separately (15, 19, 20, 23–26). The iodine non-avidity group had a poorer prognosis than the iodine avidity group for the OS (HR=3.10 [CI 1.79-5.37], P<0.01, N=646) and PFS (HR=2.93 [CI 2.18-3.95], P<0.01, N=395) (**Figures 4A, B**). A similar trend was observed for the pooled OS (**Figure 3D**).

#### 3.2.6 Metastatic nodule size

Four studies comprising 513 patients were included in the analysis of the metastatic nodule size threshold of 10mm for PFS (15, 20, 24, 26). Notably, patients with a metastatic nodule size >10mm had a significantly poor PFS than those with metastatic nodule size ≤10mm (HR=2.56 [CI 2.02-3.24], P <0.01, N=513, **Figure 4C**). One study yielded a similar result in OS (HR=2.58 [CI 1.34-4.98], P<0.001, N=113) (27). Furthermore, another study reported that patients with a negative chest CT had a better OS than those with metastatic nodule size less (HR=1.9 [CI 1.1-3.4], P<0.01) or greater than 10mm (HR=3.5 [CI 2.1-5.8], P<0.01) in 372 patients (25).

#### 3.2.7 ETE

Three studies comprising 272 patients were included in the meta-analysis of ETE for PFS (15, 20, 38). Patients with ETE had significantly poorer PFS than those without ETE (HR=2.05 [CI 1.15-3.67], P=0.02, N=271, **Figure 4D**). In particular, one study reported a significantly poor OS in patients with ETE than those without (HR=2.4 [CI 1.10-5.15], P=0.03, N=152) (15).

#### 3.2.8 Lymph node metastases

Six studies were included in the meta-analysis for LNM; three studies each for OS and three studies for PFS (15, 20, 24, 26, 33, 37). There were no significant differences in OS (HR=0.68 [CI 0.41–1.12], P=0.13, N=501) and PFS (HR=0.94 [CI 0.47-1.90], P=0.86, N=526) between patients with or without LNM (**Figures 4E** **, F**).

#### 3.2.9 Other factors

Several studies reported the primary tumor size and number of foci amongst the patients included in those studies (**Supplementary Table 2**). There were no significant differences in OS (HR=1.28 [CI 0.58 -2.80], P=0.54, N=96, **Supplementary Figure 3**) and PFS (HR=1.41 [CI 0.79-2.52], P=0.24, N=196, **Supplementary Figure 4**) between patients with primary tumor sizes over and under 40mm. Similarly, there was no significant difference in PFS between the unifocal group and the multifocal group (HR=1.81 [CI 0.77-4.25], P*=*0.18, N=154, **Supplementary Figure 5**).

#### 3.2.10 Survival at different time points

We also evaluated and conducted a meta-analysis on the OS of DTC patients with PM at different time points (**Supplementary Table 3**). The pooled 1-year OS was 95.67% (95% CI: 92.93%-98.42%). However, the OS rate declined by approximately 15% every 5 years (**Figure 3A**), with the pooled OS at 20 years declining to 51.03% (95% CI: 36.52%-65.54%). Only two studies reported the PFS of patients at different time points (26, 38). The pooled 1-year PFS was 93.20% (95% CI: 62.55%-100%), while the pooled 10-year PFS was only 38.93% (95% CI: 23.53%-56.90%).

Subgroup analyses were only conducted on the histology subtype, metastases to other organs, and iodine avidity because of the lack of relevant data. On all three factors, there were significant differences in pooled OS between groups at different time points (P<0.001, **Supplementary Table 3**). PTC patients had a better pooled OS than FTC patients. The pooled 10-year and 20-year OS of the two groups were 61.66% vs. 31.96% and 46.65% vs. 18.27%, respectively (**Figure 3B**). Notably, patients with metastases to other organs had a poorer OS than patients with lung metastases alone (**Figure 3C**). The pooled 10-year and 20-year OS of the two groups were 65.21% vs. 31.21% and 54.68% vs. 7.25%, respectively. Similarly, non-avidity patients had a worse pooled OS than iodine avidity patients (**Figure 3D**). The pooled 10-year and 20-year OS were of the two groups were 50.22% vs. 15.95% and 32.30% vs. 1.0%, respectively.

#### 3.2.11 Interstudy heterogeneity and publication bias

Variables including year of publication, sample size, region, and study quality were analyzed by meta-regression. However, no significant source of heterogeneity was identified in the meta-regression for OS of histology subtypes and metastases to other organs (P>0.05 for each, **Supplementary Table 4**). In addition, the funnel plots (**Supplementary Figure 9-11**) and Egger’s test showed no evidence of publication bias for the OS of gender (t=-0.45, P=0.66), histology subtypes (t=-0.14, P=0.89), and metastases to other organs (t=-0.96, P=0.36).

## 4 Discussion

A review of the relevant prognostic factors for DTC patients with PM was conducted to confirm their roles. To the best of the authors’ knowledge, this is the first systematic review and meta-analysis to evaluate the prognostic factors for DTCs with PM. The prognostic factors include:

### 4.1 Age

Age is an important prognostic factor and a consideration in staging for DTC patients (40). Though the age cutoff point changed to 55 years in the 8^th^ TNM (41), 45 years was still the most commonly used cutoff point for the included studies in this review. DTC patients over 45 years and with PM had a poorer OS and PFS, consistent with previous studies (40, 42, 43). Studies suggest that 55 years may be a better cutoff point to predict the recurrence and survival of DTC patients (44). However, there were no differences in PFS between the two groups (≤55 years vs. >55 years) according to this meta-analysis. Of note, the number of included studies in the analysis was small, causing a high heterogeneity (I^2^ = 68%, P=0.08). The predictive value for 55 years as a cutoff point on DTC patients with PM thus remains to be explored. Studies suggest that the elevation of the cutoff point underestimates the prognostic risk of younger patients with risk features. For instance, Adam et al. showed that LNMs were associated with a 32% increase in mortality risk for stage I patients under 45 years, indicating that the current age cutoff point might be under staging young patients, translating to their undertreatment (42). In this meta-analysis, the maximum value of HRs was observed when the cutoff point was set at 40 years (**Supplementary Figure 2**). This cutoff point may predict the prognosis better and avoid underestimating the risk for younger patients considering the coexistence of PM.

### 4.2 Gender

Though the incidence of thyroid cancer is three-fold higher in females than in males, most studies showed higher mortality (HR 1.47-2.53) and shorter PFS in males than in females (45–47). Similarly, male DTC patients with PM had a poorer OS (HR=1.36) and PFS (HR=1.10). Male DTC patients with PM should thus be subjected to more aggressive therapy than females.

### 4.3 Histological subtype

PTC and FTC account for 80%-85% and 10%-15% of DTC, respectively (48). Most studies suggest a poorer prognosis of FTC than PTC (49–51), which was also observed in patients in this review. The pooled 20-year OS of FTC was less than 50% that for PTC patients.

FTC is characterized by early vascular infiltration, making it more likely to cause distant metastases through blood flow to the bones and lungs than PTC (24, 52, 53). The proportion of DM in FTC was much higher than in PTC (4.1%-14.1% vs. 0.5%-3.6%) (54–56), an important reason why FTC has a poorer prognosis than PTC. However, the reasons for the poor survival prognosis of FTC than PTC when PM exists remains unclear. Lungs are the most common site of metastases in DTC, followed by bones, liver, and brain. Several studies revealed that the proportion of bone metastases in FTC is twice that in PTC (7-28% vs. 1.4-7%) (57–59), with incidences of liver and brain metastases in FTC also being higher than in PTC (60). Generally, these studies suggest that FTC patients have a higher probability of combining with metastases to other organs than PTC patients when lung metastases exist. For instance, Wang et al., reported that FTC patients were more likely to progress from single organ metastases (SODM) to multiple organ metastases in 5 years than PTC patients (37.5% vs. 23.7%) (61). MODM could be the reason FTC patients with PM have a poorer prognosis than PTC patients, as observed in this review. FTC combined with PM is thus a histological subtype that requires high priority.

### 4.4 Metastases to other organs

PM patients with metastases to other organs (a condition of MODM) had a significant increase in mortality risk, which was thrice higher than that of patients with PM alone (HR=3.18, P<0.01, N=1713). The pooled 20-year OS was only 7.25% for MODM patients but remained 54.68% for patients with PM alone. Several studies suggest that DTC patients with MODM have a poor prognosis than patients with SODM. In a retrospective study including 111 PTC patients with DM, Haq et al. reported a poor survival for MODM patients than patients with PM alone (CSS: HR=2.70 [CI 1.38-5.26], P=0.02) or other single sites (13). Toraih et al. analyzed 1819 DTC patients with DM from the SEER database and reported a five- to six-fold increase in mortality risk for MODM patients than SODM patients (62). A more active treatment strategy may thus be necessary for PM patients with metastases to other organs.

### 4.5 Metastatic nodule size

Radioiodine therapy is the first line of choice for DTC patients with DM (63). However, the average penetration distance of β-irradiation from ^131^I inside the tissue is only 1 mm (4), resulting in larger lesions requiring higher doses of ^131^I, poorer outcomes, and more side effects (64). The pulmonary metastatic nodules are divided into micro-nodular and macro-nodular by 10mm. In this review, the micro-nodular group had a significantly better PFS than the macro-nodular group. Yang et al. also reported a significant difference in OS between the two groups (HR=2.58, P<0.001, N=113). Furthermore, it has been reported that PM patients with negative chest CT can achieve a better prognosis (27). Song et al. reported a 15-year OS of 0% vs. 50.1% vs. 75.8% (P<0.001, N=372) when comparing the macro-nodular group and micro-nodular group with patients who had negative chest CT, respectively (25). Qiu et al. tried 5mm as the cutoff point of the PM nodule size but reported no significant differences in PFS between groups (38). The optimal cutoff point of the metastatic nodule size should thus be explored further.

### 4.6 ETE

ETE is a recognized prognostic factor for DTC and is divided into minimal extrathyroid extension (mETE) and extensive extrathyroid extension (eETE) (65, 66). A large retrospective study by Youngwirth et al. that included 241,118 cases reported that patients with ETE, especially eETE (HR=1.74, P<0.01), had a poorer survival prognosis than those without (67). In this meta-analysis, ETE remains significantly affected thyroid cancer recurrence in PM patients (HR=2.05, P=0.02, N=271). Hirsch et al. also reported that PM patients with ETE have a higher mortality risk (HR=2.4 [CI 1.1-5.4], P=0.032, N=101) (15). Of note, the studies included in this meta-analysis did not categorize ETE into mETE and eETE. The prognostic effect of mETE on DTC patients with PM remains controversial (68, 69) and should thus be further explored.

### 4.7 Primary tumor size, LNM, and multifocality

The primary tumor size is a determinant for outcome in DTC (70). Previous studies postulate that the prognosis is worse for patients with larger tumor size (71, 72). However, the results of this meta-analysis revealed no significant differences in survival and recurrence between groups with 40mm as the cutoff point. In the same line, Qiu et al. reported insignificant differences in PFS among three subgroups of PM patients divided based on the primary tumor size (<20mm vs. 20-40mm and <20mm vs. ≥40mm, P>0.05, N=47) (38). Previous studies suggest that the risk of LNM and DM increases with increasing tumor size, leading to a worse prognosis for DTC patients (73, 74). However, the prognostic impact of distant metastases overrides the impact of tumor size upon PM occurrence, explaining why the impact of tumor size is not shown in PM patients. Similarly, the meta-analysis results did not show the impact of LNM on the OS (HR=0.68, P=0.13, N=510) and PFS (HR=0.97, P=0.94, N=526) of patients with PM. Although the presence of LNM leads to an increased risk of DM and a worse prognosis, its prognostic role is masked by the impact of DM when combined with PM (75). Multifocality does not affect patient prognosis in patients with DTC and PM because of similar mechanisms (76, 77). Meta-analysis results revealed that multifocality did not affect the PFS of PM patients (HR=1.81, P=0.18, N=154). Similarly, Leite et al. also reported that multifocality did not affect the PFS of PM patients (HR=1.804, P=0.917, N=54) (34).

### 4.8 RAI therapy, targeted therapy, and immunotherapy

Radioiodine therapy is the fundamental therapy for DM patients. The uptake status of radioactive iodine plays a crucial role in the prognosis of DM patients, depending on the size of the lesion and the iodine uptake capacity (78). The loss of ability to take up radioactive iodine can be congenital or acquired during RAI therapy (79). The prognosis for the patients is poor when iodine resistance is present regardless of the cause for losing iodine uptake ability (80). The same effect applies to DTC patients with PM (4). In this meta-analysis, iodine non-avidity patients with PM had a three-fold higher risk of mortality and recurrence than avidity patients. The pooled 20-year OS of the non-avidity patients was merely 0%, while that of avidity patients remained 34.3%. This result suggested that the sensitivity to radioiodine therapy is crucial to the overall outcome for PM patients, and identifying RAI-Refractory DTC (RAIRD) has important implications for treatment decisions (81).

Radioiodine therapy in PM patients is not ideal. Studies report that one-third to one-half of DTC patients with DM have RAI refractory (82, 83). The pooled overall efficacy rate of RAI therapy is 58% in PM patients (84). It is foreseeable that quite a significant portion of PM patients have a poor prognosis if they receive only standard treatment, including surgery, RAI therapy, and TSH suppression therapy. Promisingly, the emergence of TKIs offers new treatment options for thyroid cancer and has been employed as the first-line treatment for RAIRD (85, 86). Several randomized controlled clinical trials have reported encouraging results for TKIs, such as lenvatinib and sorafenib in RAIRD (87–90). For instance, the SELECT trial showed that RAIRD patients with PM had a duration of overall response (DOR) of 29.9 months (95% CI 17.5-37.8) after treatment with lenvatinib (88). Moreover, a *post hoc* analysis of the SELECT trial showed that the lenvatinib group had a longer median OS (44.7 months vs. 33.1 months, P<0.01, N=392) than the placebo group in patients with PM lesions ≥10mm (30). Previous studies postulate that combining TKIs and immune checkpoint inhibitors (ICIs) could improve RAIRD treatment (91). A recent study reported better clinical benefit on PFS when lenvatinib was combined with pembrolizumab for RAIRD than using lenvatinib alone (92). However, further studies are needed to ascertain the effectiveness of these combinations in DTC patients with PM. The use of TKIs and ICIs in DTCs is still at an early stage and is thus important to identify the right target and the optimal treatment time (4, 86). Current ATA guidelines recommend using TKIs in patients with metastases, rapid progression, and symptomatic and life-threatening diseases. However, some researchers argue that patients potentially miss the optimal treatment time before meeting these requirements (86). This meta-analysis suggested that DTC patients with PM have a poor prognosis, especially those with FTC, multi-organ metastases, or iodine non-avidity (**Figure 3**). It is thus worth considering and exploring whether these patients should be treated with TKIs more aggressively before significant tumor progression.

### 4.9 Other prognostic factors

Serum Tg is the main biochemical tumor marker used to detect postoperative recurrence in patients with DTC (93). Previous studies postulate that serum Tg levels ≥50μg/L are associated with poorer PFS and OS in patients with DTC (94). However, the prognostic role of serum Tg in DTC patients with PM remains to be further investigated. Chopra et al. reported a poorer DFS in the ≥50μg/L group than the <50μg/L group (10 years DFS: 70% vs. 35%, HR=4.59 [CI 1.02-20.62], P=0.047, N=42) (21). In contrast, Chen et al. reported no significant differences in PFS between the two groups (HR=4.37 [CI 0.59-32.47], P=0.15, N=103) (20). Tg doubling time (TgDT) is a more predictable factor in PM patients than serum Tg levels (95). Miyauchi et al. reported that the 10-year cancer-specific survival (CSS) in PTC patients was only 50% when TgDT was <1 year, which was significantly worse than in patients with TgDT of 1-3 years (CSS: 95%) or >3 years (CSS: 100%) (96). However, no studies have reported the role of TgDT in PM patients.

Serum anti-thyroglobulin antibody (TgAb) level has been reported to predict persistent or recurrent diseases in DTC patients (97, 98). However, there are no sufficient studies to ascertain whether TgAb can be used as a prognosis indicator in PM patients. Qiu et al. evaluated the association between TgAb levels and the prognosis of DTC patients with PM and reported insignificant differences in PFS at 5 years (P=0.725) and 10 years (P=0.739) between different TgAb level groups in 47 patients (38).

BRAF^V600E^ mutations, RAS mutations, and TERT promoter mutations are enriched in primary tumors of DTC with DM (99). Numerous studies confirm their association with aggressive biological behavior and worse prognosis (100–104). Xing et al. reported a significantly higher mortality rate in patients with BRAF^V600E^ mutation in primary tumor than unmutated DTC patients with DM (51.5% vs. 18.2%, P<0.001, N=1849) (105), attributed to the RAI resistance caused by BRAF^V600E^ mutation (106). Song et al. reported that the recurrence risks for DTC patients with TERT promoter mutations in the primary tumor were 5.79 times and 3.6 times higher than those without mutations among ATA high-risk patients and stage III/IV patients, respectively (107). To date, no studies have evaluated the prognostic impact of the gene mutations in the primary tumor on DTC patients with PM and their impact on metastatic lesions.

### 4.10 Strengths and limitations of the study

This study is characterized by several strengths. It follows the recent guidelines on the meta-analysis of prognostic factor studies, which recommends an evaluation of the overall quality and reliability of the pooled results for clinical guidance using the GRADE approach. The study fully used the data from the included studies and systematically reviewed the prognostic factors for DTC patients with DM for the first time. The systematic review and meta-analysis revealed important prognostic factors for DTC patients with PM, such as histological subtype, extrapulmonary metastases, and iodine avidity.

Nevertheless, this study was limited by several factors. First, all the included studies were retrospective cohort studies, and thus most of them had confounding factors, including missing baseline and prognostic survival data because of the lack of a pre-established study protocol. These factors could have potentially affected the identification and assessment of prognostic survival factors (108). Second, high statistical heterogeneity was observed in most meta-analysis, but the source of heterogeneity was not well recognized. Since the included studies fewer than ten, meta-regression was not performed on most prognostic factors. The large time span (1985–2019) that induces confounding factors may explain part of the heterogeneity, however, unexplained heterogeneity was inevitable because the study was a meta-analysis and review of prognostic survival factors (32). Additionally, due to insufficient data, most individual meta-analyses included a small number of studies, and some important prognostic factors could not be performed. Finally, because of the lack of literature available, the prognostic impact of molecular biomarkers was not thoroughly discussed here. Hopefully, future research in this field will fill some of these gaps.

## 5 Conclusions

In this systematic review and meta-analysis, we evaluated the prognostic factors for DTC patients with PM based on the clinicopathological, therapeutic, and biochemical aspects. We found that these patients had a poor overall prognosis, especially those with FTC, metastases to other organs, or iodine non-avidity. In additional, emerging therapies have yielded encouraging results in these patients but still requires further investigation. These findings will help in better prognostic risk stratification and clinical management in DTC patients with PM.

## Data availability statement

The original contributions presented in the study are included in the article/**Supplementary Material**. Further inquiries can be directed to the corresponding author.

## Author contributions

Protocol and design of study: HZ, C-HL,X-YL,YC. Acquisition and/or management of data: HZ, C-HL, YZ. Analysis and/or interpretation of data: HZ, C-HL, Y-YL. Drafting and revising the manuscript: HZ, X-YL, L-YZ, Y-WL, H-FL, and Y-SL. All authors contributed to the article and approved the submitted version.
