# Supplementary material for: Survival prognostic factors for differentiated thyroid cancer patients with pulmonary metastases: A systematic review and meta-analysis
Source: Front Oncol. 2022 Dec 15;12:990154. doi: 10.3389/fonc.2022.990154 (PMC9798085; doi:10.3389/fonc.2022.990154)
Supplement: Supplementary file 1 [file DataSheet_1.docx]

Supplementary Material

# Supplementary Data

**S1. Searching strategy**

**Pubmed**

| #1 | ("DTC"[Title/Abstract] OR "differentiated thyroid cancer*"[Title/Abstract] OR "differentiated thyroid carcinoma*"[Title/Abstract] OR "differentiated thyroid neoplasm*"[Title/Abstract]) |
| --- | --- |
| #2 | "thyroid cancer, papillary" [MeSH Terms] |
| #3 | (((((((((((((((((("cancer papillary thyroid"[Title/Abstract] OR "cancers papillary thyroid"[Title/Abstract]) OR "papillary thyroid cancer"[Title/Abstract]) OR "papillary thyroid cancers"[Title/Abstract]) OR "thyroid cancers papillary"[Title/Abstract]) OR "thyroid carcinoma papillary"[Title/Abstract]) OR "carcinoma papillary thyroid"[Title/Abstract]) OR "carcinomas papillary thyroid"[Title/Abstract]) OR "papillary thyroid carcinomas"[Title/Abstract]) OR "thyroid carcinomas papillary"[Title/Abstract]) OR "papillary carcinoma of thyroid"[Title/Abstract]) OR "papillary thyroid carcinoma"[Title/Abstract]) OR "familial nonmedullary thyroid cancer"[Title/Abstract]) OR "nonmedullary thyroid carcinoma"[Title/Abstract]) OR (((("Carcinoma"[MeSH Terms] OR "Carcinoma"[All Fields]) OR "Carcinomas"[All Fields]) OR "carcinoma s"[All Fields]) AND "nonmedullary thyroid"[Title/Abstract])) OR (((("Carcinoma"[MeSH Terms] OR "Carcinoma"[All Fields]) OR "Carcinomas"[All Fields]) OR "carcinoma s"[All Fields]) AND "nonmedullary thyroid"[Title/Abstract])) OR "nonmedullary thyroid carcinomas"[Title/Abstract]) OR "thyroid carcinoma nonmedullary"[Title/Abstract]) OR ((((("thyroid neoplasms"[MeSH Terms] OR ("Thyroid"[All Fields] AND "neoplasms"[All Fields])) OR "thyroid neoplasms"[All Fields]) OR ("Thyroid"[All Fields] AND "Carcinomas"[All Fields])) OR "thyroid carcinomas"[All Fields]) AND "Nonmedullary"[Title/Abstract])) |
| #4 | #2 OR #3 |
| #5 | "adenocarcinoma, follicular" [MeSH Terms] |
| #6 | "adenocarcinomas follicular"[Title/Abstract] OR "follicular adenocarcinoma"[Title/Abstract] OR "follicular adenocarcinomas"[Title/Abstract] OR "thyroid carcinoma follicular"[Title/Abstract] OR "follicular thyroid carcinoma"[Title/Abstract] OR "carcinoma follicular thyroid"[Title/Abstract] OR "carcinomas follicular thyroid"[Title/Abstract] OR "follicular thyroid carcinomas"[Title/Abstract] OR "thyroid carcinomas follicular"[Title/Abstract] |
| #7 | #5 OR #6 |
| #8 | #1 OR #4 OR #7 |
| #9 | ((("lung"[MeSH Terms] OR "lung"[All Fields] OR "pulmonary"[All Fields]) AND "metastas*"[All Fields]) OR ("distant"[All Fields] AND "metastas*"[All Fields])) |
| #10 | "follow up"[All Fields] OR "prognos*"[All Fields] |
| #11 | 1900/01/01:2021/02/28[Date - Publication] |
| #12 | #8 AND #9 AND#10 #11 |

**Cochrane library**

| #1 | MeSH descriptor: [Adenocarcinoma, Follicular] explode all trees |
| --- | --- |
| #2 | MeSH descriptor: [Thyroid Cancer, Papillary] explode all trees |
| #3 | (Adenocarcinomas, Follicular):ti,ab,kw |
| #4 | (Follicular Adenocarcinoma):ti,ab,kw |
| #5 | (Follicular Adenocarcinomas):ti,ab,kw |
| #6 | (Thyroid Carcinoma, Follicular):ti,ab,kw |
| #7 | (Follicular Thyroid Carcinoma):ti,ab,kw |
| #8 | (Carcinoma, Follicular Thyroid):ti,ab,kw |
| #9 | (Carcinomas, Follicular Thyroid):ti,ab,kw |
| #10 | (Follicular Thyroid Carcinomas):ti,ab,kw |
| #11 | (Thyroid Carcinomas, Follicular):ti,ab,kw |
| #12 | (Cancer, Papillary Thyroid):ti,ab,kw |
| #13 | (Cancers, Papillary Thyroid):ti,ab,kw |
| #14 | (Papillary Thyroid Cancer):ti,ab,kw |
| #15 | (Papillary Thyroid Cancers):ti,ab,kw |
| #16 | (Thyroid Cancers, Papillary):ti,ab,kw |
| #17 | (Thyroid Carcinoma, Papillary):ti,ab,kw |
| #18 | (Carcinoma, Papillary Thyroid):ti,ab,kw |
| #19 | (Carcinomas, Papillary Thyroid):ti,ab,kw |
| #20 | (Papillary Thyroid Carcinomas):ti,ab,kw |
| #21 | (Thyroid Carcinomas, Papillary):ti,ab,kw |
| #22 | (Papillary Carcinoma Of Thyroid):ti,ab,kw |
| #23 | (Papillary Thyroid Carcinoma):ti,ab,kw |
| #24 | (Familial Nonmedullary Thyroid Cancer):ti,ab,kw |
| #25 | (Nonmedullary Thyroid Carcinoma):ti,ab,kw |
| #26 | (Carcinoma, Nonmedullary Thyroid):ti,ab,kw |
| #27 | (Carcinomas, Nonmedullary Thyroid):ti,ab,kw |
| #28 | (Nonmedullary Thyroid Carcinomas):ti,ab,kw |
| #29 | (Thyroid Carcinoma, Nonmedullary):ti,ab,kw |
| #30 | (Thyroid Carcinomas, Nonmedullary):ti,ab,kw |
| #31 | (DTC):ti,ab,kw |
| #32 | (differentiated thyroid cancer*):ti,ab,kw |
| #33 | (differentiated thyroid carcinoma*):ti,ab,kw |
| #34 | (differentiated thyroid neoplasm*):ti,ab,kw |
| #35 | #1 OR #2 OR #3 OR #4 OR #5 OR #6 OR #7 OR #8 OR #9 OR #10 OR #11 OR #12 OR #13 OR #14 OR #15 OR #16 OR #17 OR #18 OR #19 OR #20 OR #21 OR #22 OR #23 OR #24 OR #25 OR #26 OR #27 OR #28 OR #29 OR #30 OR #31 OR #32 OR #33 OR #34 |
| #36 | (lung* metastas*):ti,ab,kw |
| #37 | (pulmonary metastas*):ti,ab,kw |
| #38 | (distant metastas*):ti,ab,kw |
| #39 | #36 OR #37 OR #38 |
| #40 | (prognos*) OR (follow-up) |
| #41 | #35 AND #39 AND #40 |

**Scopus**

| #1 | TITLE-ABS-KEY(Follicular Thyroid Carcinoma*) OR TITLE-ABS-KEY(Follicular Thyroid Cancer*) OR TITLE-ABS-KEY(Papillary Thyroid Carcinoma*) OR TITLE-ABS-KEY(Papillary Thyroid Cancer*) OR TITLE-ABS-KEY(Differentiated Thyroid Cancer*) OR TITLE-ABS-KEY(Differentiated Thyroid Carcinoma*) OR TITLE-ABS-KEY(Differentiated Thyroid Neoplasm*) |
| --- | --- |
| #2 | TITLE-ABS-KEY(lung* metastas*) OR TITLE-ABS-KEY(pulmonary metastas*) OR TITLE-ABS-KEY(distant metastas*) |
| #3 | 'Follow-up' OR 'prognos*' |
| #4 | #1 AND #2 And #3 |

**Embase**

| #1 | 'differentiated thyroid cancer'/exp |
| --- | --- |
| #2 | 'thyroid papillary carcinoma'/exp |
| #3 | 'adenocarcinomas, follicular':ab,ti OR 'follicular adenocarcinoma':ab,ti OR 'follicular adenocarcinomas':ab,ti OR 'thyroid carcinoma, follicular':ab,ti OR 'follicular thyroid carcinoma':ab,ti OR 'carcinoma, follicular thyroid':ab,ti OR 'carcinomas, follicular thyroid':ab,ti OR 'follicular thyroid carcinomas':ab,ti OR 'thyroid carcinomas, follicular':ab,ti |
| #4 | 'thyroid follicular carcinoma'/exp |
| #5 | 'adenocarcinomas, follicular':ab,ti OR 'follicular adenocarcinoma':ab,ti OR 'follicular adenocarcinomas':ab,ti OR 'thyroid carcinoma, follicular':ab,ti OR 'follicular thyroid carcinoma':ab,ti OR 'carcinoma, follicular thyroid':ab,ti OR 'carcinomas, follicular thyroid':ab,ti OR 'follicular thyroid carcinomas':ab,ti OR 'thyroid carcinomas, follicular':ab,ti |
| #6 | #1 OR #2 OR #3 OR #4 OR #5 |
| #7 | 'lung metastasis'/exp |
| #8 | 'lung metastas*':ab,ti OR 'pulmonary metastas*':ab,ti |
| #9 | 'distant metastasis'/exp |
| #10 | 'distant metastas*':ab,ti |
| #11 | #7 OR #8 OR #9 OR #10 |
| #12 | 'follow up' OR 'prognos*' |
| #13 | #6 AND 11 AND 12 |

**China national knowledge infrastructure** (Translated from Chinese)

| SU=(differentiated thyroid cancer + papillary thyroid cancer + follicular thyroid cancer) AND TKA=( pulmonary metastas* + distant metastas* ) AND prognos* |
| --- |

**Wangfang database** (Translated from Chinese)

| Topic:( differentiated thyroid cancer OR papillary thyroid cancer OR follicular thyroid cancer) And (Title or Key word:(pulmonary metastas* or lung metastas* or distant metastas*)) OR Abstract:( pulmonary metastas* or lung metastas* or distant metastas*) and All Field:(prognos* ) |
| --- |

**VIP database** (Translated from Chinese)

| ((M=(differentiated thyroid cancer+differentiated thyroid carcinoma) + R=(differentiated thyroid cancer+differentiated thyroid carcinoma)) + (M=(papillary carcinoma of thyroid+papillary thyroid cancer+papillary thyroid carcinoma+papillary thyroid carcinomas+thyroid papillary carcinoma) + R=(papillary carcinoma of thyroid+papillary thyroid cancer+papillary thyroid carcinoma+papillary thyroid carcinomas+thyroid papillary carcinoma)) + (M=(follicular thyroid carcinoma) + R=(follicular thyroid carcinoma)))* (M=(pulmonary metastas*+ lung metastas* + distant metastas*) + R=(pulmonary metastas*+ lung metastas*+ distant metastas*)) * U=prognos* |
| --- |

# Supplementary Figures and Tables

## Supplementary Figures


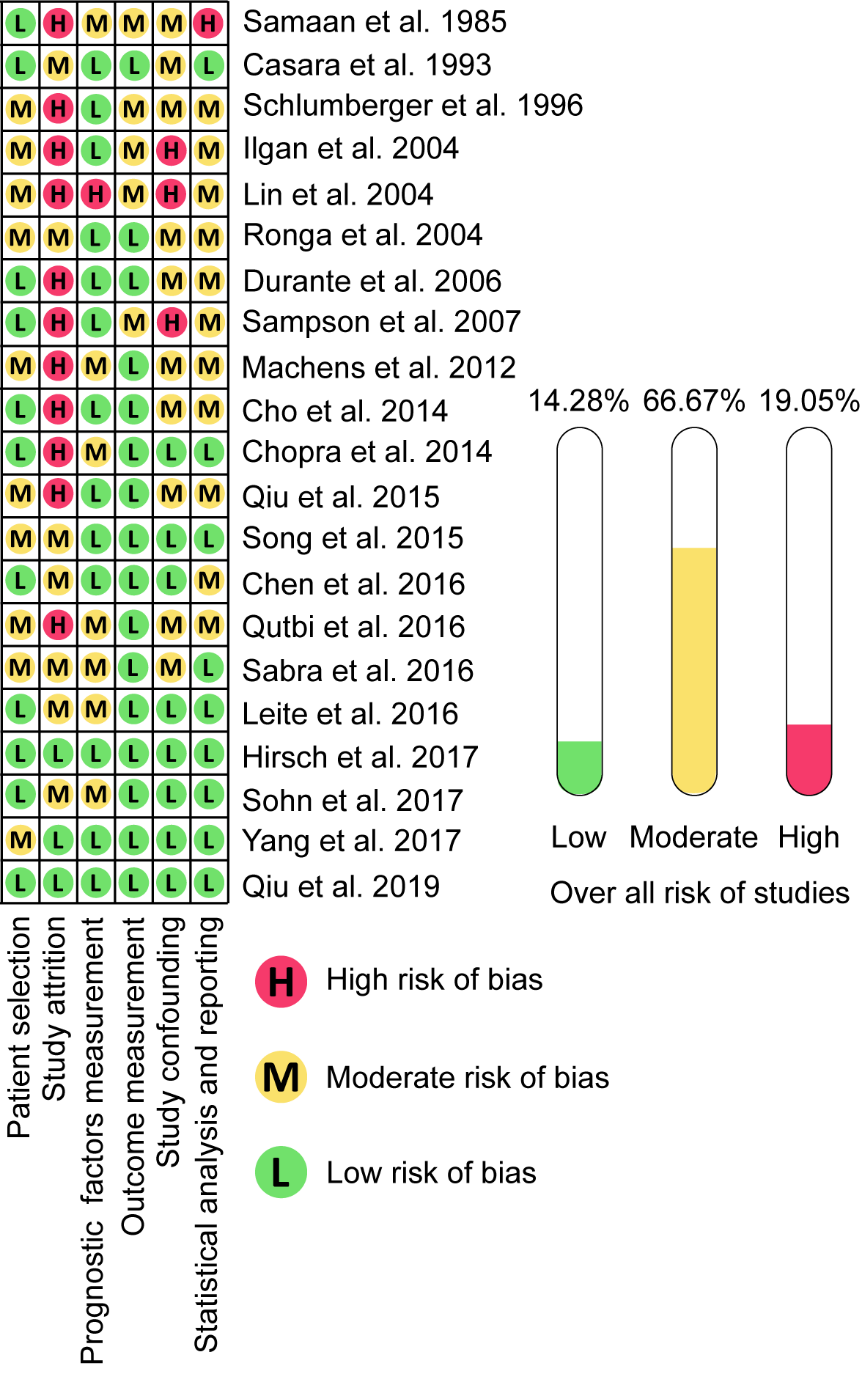


**Supplementary Figure 1.** Evaluating applicability and risk of bias of primary studies with QUIPS checklist.

**

**

**Supplementary Figure 2.** Hazard ratio trend by age.





**Supplementary Figure 3.** Forest plot for the associate of age with OS (>40 years vs. ≤40 years).


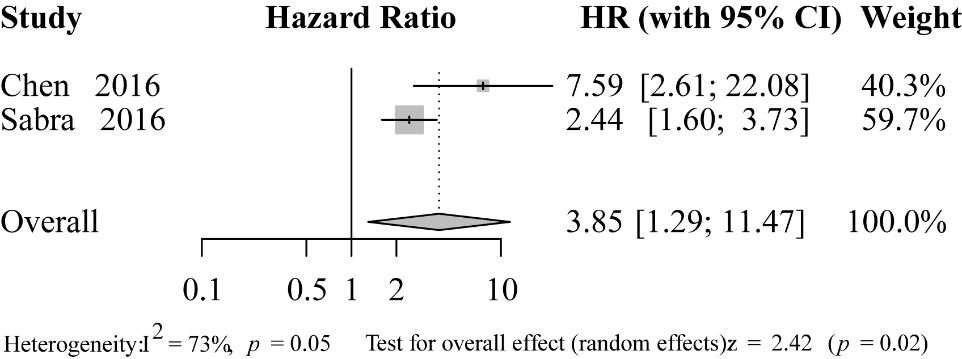


**Supplementary Figure 4.** Forest plots for the association of age with PFS (≥45years vs. <45 years).





**Supplementary Figure 5.** Forest plots for the association of age with PFS (≥55years vs. <55 years).


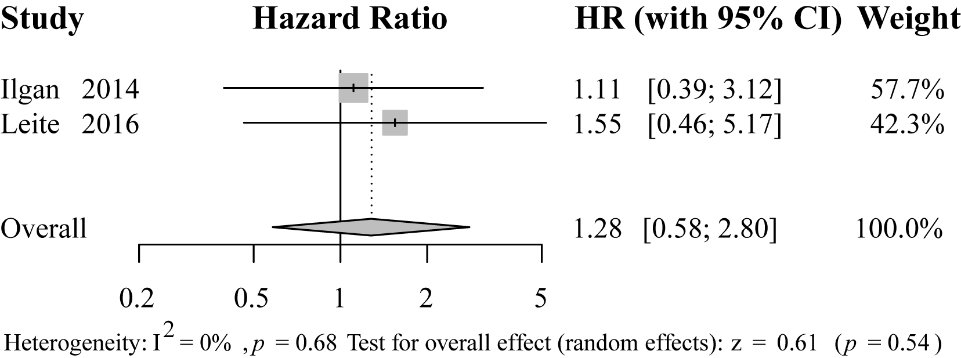


**Supplementary Figure 6.** Forest plots for the association of primary tumor size with OS

(≥40mm vs. <40mm).


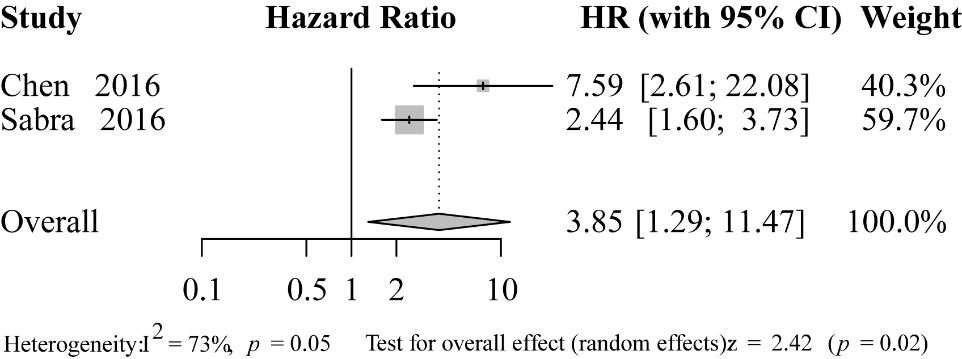


**Supplementary Figure 7.** Forest plots for the association of primary tumor size with PFS

(≥40mm vs. <40mm).


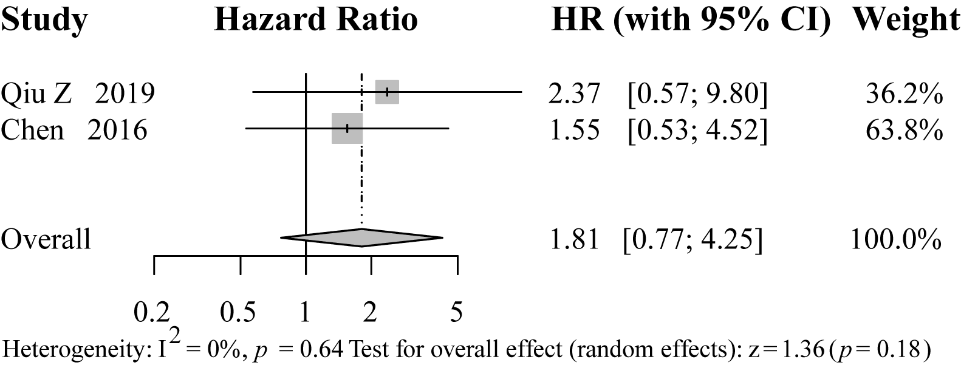


**Supplementary Figure 8.** Forest plots for the association of multifocality with PFS (multifocal vs unifocal).


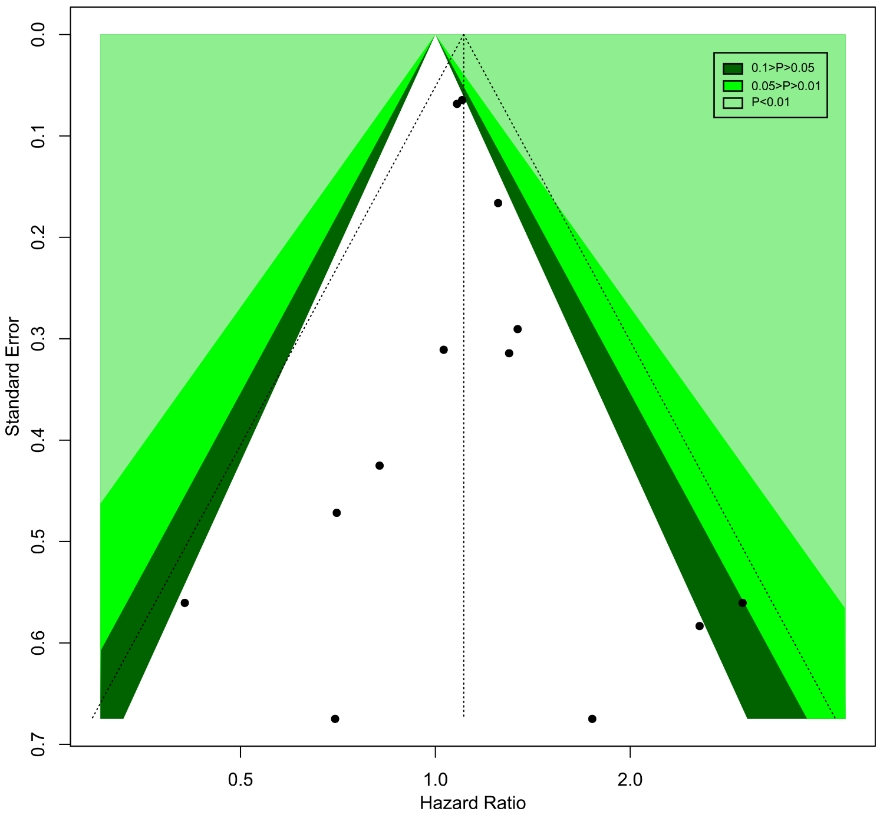


**Supplementary Figure 9.** Funnel plot of gender for OS.


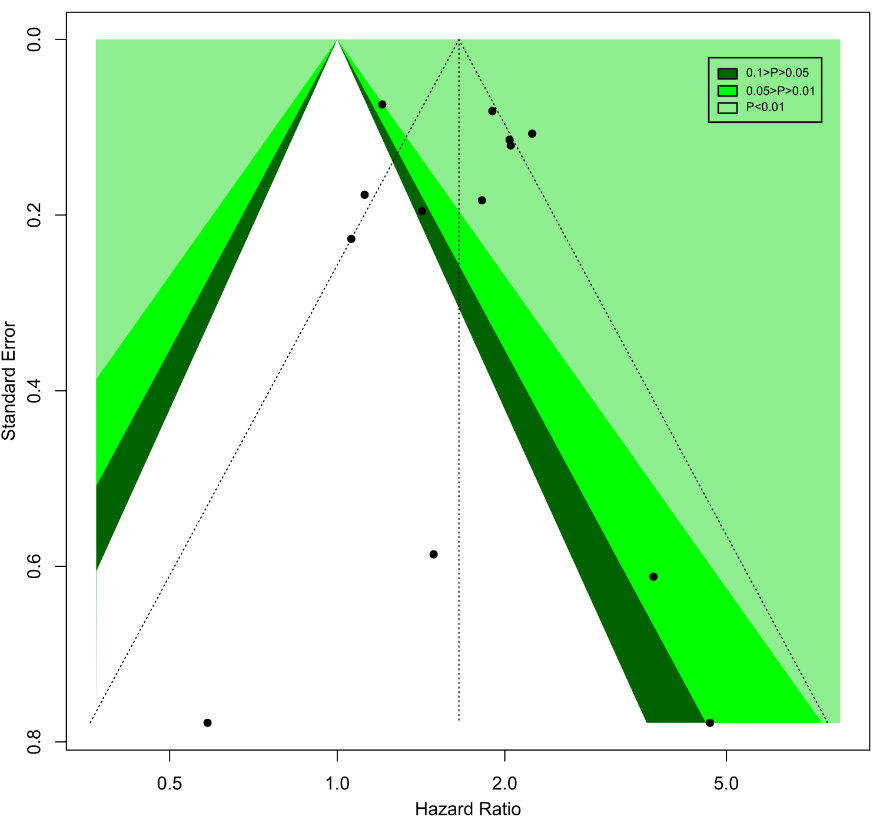


**Supplementary Figure 10.** Funnel plot of histological subtype for OS.


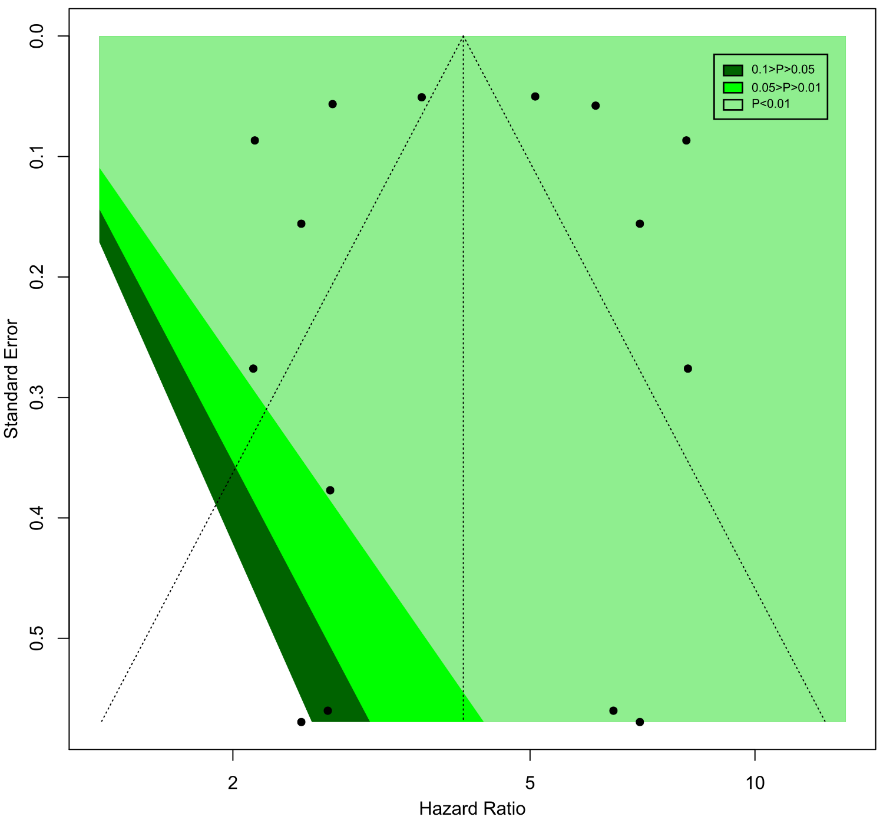


**Supplementary Figure 11.** Funnel plot of metastases to other organs for OS.

## Supplementary Tables

**Supplementary Table 1.** Characteristics for studies included in the present meta-analysis

| First Author | Published Year | Study  Design | Size* | Male | Mean Age (mean ± SD) | Mean follow-up time (year) | Continent of study | Sites |
| --- | --- | --- | --- | --- | --- | --- | --- | --- |
| Samaan | 1985 | R | 101 | 47.5% | 46.0 ± 19.0 | NA | North America | Single centre |
| Casara | 1993 | R | 134 | 35.8% | 50.9 ±18.2 | 6.6 | Europe | Single centre |
| Schlumberger | 1996 | R | 284 | NA | NA | 8±6 | Europe | Single centre |
| Ilgan | 2004 | R | 42 | 64.3% | 36.0 ± 19.0 | 6.4 | Asia | Single centre |
| Lin | 2004 | R | 70 | 28.6% | 55.6 | 6.6 | Asia | Single centre |
| Ronga | 2004 | R | 96 | 28.2% | 45.9±17.9 | 16.5±11.5 | Europe | Single centre |
| Durante | 2006 | R | 305 | 38.1% | NA | NA | Europe | Single centre |
| Sampson | 2007 | R | 22 | 31.0% | 68.0 | 3.5 | North America | Single centre |
| Machens | 2012 | R | 79 | NA | NA | NA | Europe | Single centre |
| Cho | 2014 | R | 152 | 21.7% | 45.6 | 12 | Asia | Single centre |
| Chopra | 2014 | R | 200 | 31.0% | 46.3 | 5.1 | Asia | Multi-centre |
| Qiu | 2015 | R | 25 | 36.0% | 46.0 ±15.8 | 9.6 | Asia | Single centre |
| Song | 2015 | R | 372 | 36.02 | 54.3 ±16.1 | 5.0±3.4 | Asia | Single centre |
| Chen | 2016 | R | 107 | 32.7% | 41.0 | 2 | Asia | Single centre |
| Qutbi | 2016 | R | 75 | 20% | 43.8 ± 18.5 | 3.3 | Asia | Single centre |
| Sabra | 2016 | R | 199 | 47% | 50.0 ± 20.0 | 6.9 | North America | Single centre |
| Leite | 2016 | R | 54 | 35.2% | 51.6 ± 16.8 | NA | South America | Single centre |
| Hirsch | 2017 | R | 118 | 45.8% | 51.0 ± 20.0 | 8.2 | Asia | Single centre |
| Sohn | 2018 | R | 89 | 32.6% | 46.1 ± 17.8 | 7.0 | Asia | Single centre |
| Yang | 2017 | R | 151 | 43.0% | NA | 11.2 | Asia | Single centre |
| Qiu | 2019 | R | 47 | 29.8% | 39.6 ± 15.4 | 6.9 | Asia | Single centre |

*: pulmonary metastasis cases, NA = not available, R = retrospective

**Supplementary Table 2.** The data that not included in meta-analysis

| Studies | Prognostic factors | Comparison | HR (95%CI) | P value | Endpoint |
| --- | --- | --- | --- | --- | --- |
| Chen et al. 2016 | histology | PTC vs. FTC | 1.32 (0.54 – 3.21) | 0.54 | PFS |
| Chen et al. 2016 | other metastatic foci | with vs. without | 2.38 (1.10 – 5.15) | 0.03 | PFS |
| Hirsch et al. 2017 | extra-thyroid extension | Yes vs. No | 2.40 (1.10 – 5.40) | NA | OS |
| Ilgan et al. 2004 | Age | >25 vs. ≤25 | 2.36 (1.67 – 3.04) | <0.01 | OS |
| Leite et al. 2016 | multifocality | Yes vs. No | 1.80 (0.24 – 4.87) | 0.92 | OS |
| Leite et al. 2016 | Frequency of ^131^I treatment | >2 vs. ≤2 | 0.85 (0.17 – 4.33) | 0.85 | OS |
| Qiu et al. 2019 | PM nodule size (mm) | 5-10 vs. <5 | 1.01 (0.16 – 6.48) | 0.99 | PFS |
| Qiu et al. 2019 | PM nodule size (mm) | ≥10 vs. <5 | 5.82 (0.40 – 84.19) | 0.20 | PFS |
| Qiu et al. 2019 | Primary tumor size (mm) | 20-40 vs. <20 | 1.27 (0.16 – 10.16) | 0.82 | PFS |
| Qiu et al. 2019 | Primary tumor size (mm) | ≥40 vs. <20 | 2.34 (0.50- 8.19) | 0.88 | PFS |
| Qiu et al. 2019 | Hashimoto’s thyroiditis | Yes vs. No | 3.189 (0.34 – 5.00) | 0.06 | PFS |
| Qiu et al. 2019 | local recurrence before  the diagnosis of lung  metastases | Yes vs. No | 0.38 (0.03 – 5.48) | 0.48 | PFS |
| Qiu et al. 2019 | TgAb level (IU/mL) | 1000-4000 vs. <1000 | 0.023 (0.002 – 0.28) | 0.053 | PFS |
| Qiu et al. 2019 | TgAb level (IU/mL) | ≥4000 vs. <1000 | 0.09 (0.07 – 11.13) | 0.94 | PFS |
| Song et al. 2015 | PM nodule size(mm) | >10 vs. Negative | 3.5 (2.10 – 5.80) | < 0.01 | OS |
| Song et al. 2015 | PM nodule size(mm) | ≤10 vs. Negative | 1.90 (1.10 – 3.40) | < 0.001 | OS |
| Yang et al. 2017 | PM nodule size(mm) | ≥10 vs. ＜10 | 2.58 (1.34 – 4.98) | < 0.001 | OS |
| Yang et al. 2017 | Frequency of ^131^I treatment | ＜5 vs. ≥5 | 2.55 (1.40 – 6.43) | < 0.01 | OS |
| Chen et al. 2016 | sTg(μg/L) | <50 vs. ≥50 | 4.37 (0.59-32.47) | 0.15 | PFS |
| Chopra et al. 2014 | sTg(μg/L) | <50 vs. ≥50 | 4.59 (1.02–20.62) | 0.047 | DFS |

**Supplementary Table 3.** Pooled overall survival for DTC patients with PM at different time points.

| **Outcome** | | **Number of Study** | **Heterogeneity (I^2^ %, P)** | **Estimate (95% CI)%** | **P value** |
| --- | --- | --- | --- | --- | --- |
| **Overall** | | | | |  |
| 1 year | | 9 | 87.8, <0.001 | 95.24 [93.61 – 96.66] | - |
| 3 years | | 8 | 81.2, <0.001 | 88.46 [83.13 – 93.78] | - |
| 5 years | | 9 | 86.1, <0.001 | 78.36 [70.16 – 86.56] | - |
| 10 years | | 8 | 88.5, <0.001 | 64.86 [51.74 – 77.97] | - |
| 15 years | | 6 | 85.9, <0.001 | 56.57 [37.89 – 75.25] | - |
| 20 years | | 5 | 85.8, <0.001 | 51.03 [36.52 – 65.54] | - |
| **Histological subtype** | | | | |  |
| 1 year | PTC | 3 | 81.1, <0.001 | 95.61 [86.86 – 99.93] | <0.001 |
|  | FTC | 2 | 0, 0.38 | 82.48 [74.75 – 90.21] |  |
| 3 years | PTC | 3 | 69.6, 0.04 | 86.37 [78.08 – 94.67] | <0.001 |
|  | FTC | 2 | 0, 0.57 | 64.34 [53.98 – 74.71] |  |
| 5 years | PTC | 3 | 66.5, 0.05 | 77.18 [66.07 – 88.29] | <0.001 |
|  | FTC | 2 | 0, 0.97 | 52.78 [41.37 – 64.18] |  |
| 10 years | PTC | 3 | 0, 0.60 | 61.66 [53.72 – 69.59] | <0.001 |
|  | FTC | 2 | 0, 0.74 | 31.96 [19.72 – 44.20] |  |
| 15 years | PTC | 2 | 37.9, 0.20 | 53.72 [41.05 – 66.40] | <0.001 |
|  | FTC | 2 | 0, 0.56 | 26.04 [13.08 – 38.99] |  |
| 20 years | PTC | 2 | 0, 0.34 | 46.65 [35.60 – 57.69] | <0.001 |
|  | FTC | 2 | 0, 0.67 | 18.27 [4.51 – 32.03] |  |
| **Metastases to other organs** | | | | |  |
| 1 years | without | 4 | 0, 0.44 | 95.86 [90.71 – 99.26] | <0.001 |
|  | with | 5 | 69.3, 0.01 | 90.24 [84.64 – 94.74] |  |
| 3 years | without | 4 | 73.4, 0.01 | 87.04 [80.33 – 93.74] | <0.001 |
|  | with | 5 | 48.8, 0.10 | 74.36 [64.53 – 84.20] |  |
| 5 years | without | 4 | 82.4, <0.001 | 80.77 [70.23 – 91.31] | <0.001 |
|  | with | 5 | 73.8, <0.001 | 58.82 [42.54 – 75.10] |  |
| 10 years | without | 4 | 0.1, 0.39 | 65.21 [59.37 – 71.05] | <0.001 |
|  | with | 5 | 81.2, <0.001 | 31.21 [11.55 – 50.86] |  |
| 15 years | without | 4 | 14.3, 0.32 | 59.69 [51.50 – 67.87] | <0.001 |
|  | with | 4 | 97.6, <0.001 | 13.54 [0.14– 40.66] |  |
| 20 years | without | 4 | 0, 0.44 | 54.68 [45.35 – 64.02] | <0.001 |
|  | with | 4 | 96.7, <0.001 | 7.25 [0 – 9.12] |  |
| **Iodine avidity** | | | | |  |
| 1 years | avidity | 2 | 46.1, 0.17 | 92.28 [88. 38 –95.47] | <0.001 |
|  | non-avidity | 2 | 0, 0.76 | 70.92 [61.51 – 80.33] |  |
| 3 years | avidity | 2 | 92.0, <0.001 | 76.00 [56.40 – 95.60] | <0.001 |
|  | non-avidity | 2 | 0, 0.73 | 36.51 [25.68 – 47.34] |  |
| 5 years | avidity | 2 | 88.7, <0.001 | 66.50 [45.92 – 87.08] | <0.001 |
|  | non-avidity | 2 | 0, 1.00 | 16.00 [6.59 – 25.41] |  |
| 10 years | avidity | 2 | 83.9, 0.01 | 50.22 [27.68 – 72.75] | <0.001 |
|  | non-avidity | 2 | 0,0.9628 | 15.95 [9.36 – 23.79] |  |
| 15 years | avidity | 2 | 17.4, 0.27 | 41.23 [30.74 – 51.71] | <0.001 |
|  | non-avidity | 2 | 0, 0.69 | 2.27 [0. 05 – 6.49] |  |
| 20 years | avidity | 2 | 0, 0.57 | 32.30 [21.06 – 43.54] | NA |
|  | non-avidity | 1 | NA, NA | NA* |  |

*: Only one study reported the OS result of non-avidity at 20 years, which reported as 1%

**Supplementary Table 4.** The result of meta-regression.

| **Prognostic factors** | **Coef** | **P value** | **95% CI** |
| --- | --- | --- | --- |
| Histology subtypes |  |  |  |
| Year of publication (year< 2010 vs. year> = 2010) | 0.2315 | >0.99 | -0.4543 – 0.4531 |
| Sample size (<100 cases vs. >=100 cases) | 0.2939 | 0.56 | -0.7474 – 0.4046 |
| Region (Eastern vs. Western) | 0.0926 | 0.96 | -0.1865 – 0.1763 |
| Study quality (low risk vs. not low risk) | 0.3264 | 0.35 | -0.923 – 0.3373 |
| Metastases to other organs |  |  |  |
| Year of publication (year< 2010 vs. year> = 2010) | 0.3795 | 0.86 | -0.7987 – 0.6889 |
| Sample size (<100 cases vs. >=100 cases) | 0.4383 | 0.51 | -0.6382 – 1.0799 |
| Region (Eastern vs. Western) | 0.4341 | 0.34 | -0.4376 – 1.2641 |
| Study quality (low risk vs. not low risk) | 0.4020 | 0.16 | -0.7250 – 0.8510 |
